# Using of intensity analysis approach in Benin coastal zone (West Africa) to assess land use/land cover change for further decision making

**DOI:** 10.1016/j.heliyon.2022.e12384

**Published:** 2022-12-17

**Authors:** SÃüna Donalde DolorÃüs Marguerite DÃ©guÃ©non, O.N. Fabrice BaguÃ©rÃ©, Oscar Teka, Denis Worlanyo Aheto, Brice Sinsin

**Affiliations:** a Laboratory of Applied Ecology, Faculty of Agronomic Sciences, University of Abomey-Calavi, Benin; b Laboratory of Cartography, Faculty of Geography, University of Abomey-Calavi, Benin; c Laboratory of Applied Ecology, Faculty of Agronomic Sciences, University of Abomey-Calavi, Benin; 01 BP 526, Cotonou, Benin; d Africa Centre of Excellence in Coastal Resilience - Centre for Coastal Management, Department of Fisheries and Aquatic Sciences, School of Biological Sciences, University of Cape Coast, Ghana

**Keywords:** Coastal zone, Urbanization, LU/LC change, Intensity analysis, Benin

## Abstract

Coastal areas are fruitful environments with a complex diversity of ecosystems. These areas are very sensitive and therefore, changes in the region of interest (ROI) require special attention due to the consequences. The changes observed in the coastal zone of Benin, such as: coastal erosion, the decrease of mangrove ecosystems and its consequences, and the pressure on agricultural land, have motivated this study, which aims to assess land use land cover in the coastal zone of Benin in order to better anticipate the phenomena of loss and fragmentation of ecosystems and to provide guidelines for policy-making. To achieve this objective, remote sensing and field surveys were used. Spot and Landsat satellite images of the years 1991–2006 and 2021 have been uploaded to regards. cnes.fr and USGS. Direct field observations and group discussions to determine the driving forces behind the changes were conducted. Supervised classification using the Maximum Likelihood approach of ENVI software was used and QGIS 3.16 to process the data. Significant changes have been observed in the coastal zone our study area over the past thirty years. During the period 1991–2006, palm fields constituted the largest land use with 84786 ha or 28.9% of the total area. This occupation will decrease over time to reach 66773.2 ha in 2006 (22.7% of the total area) and 27406.5 ha or 7.2% of the total area in 2021. Classes such as Mosaic of crop and out of crop, dense forest have experienced the same evolution while the opposite trend is observed in built-up areas. From 11543 ha or 3.9% of the total area in 1991, this class has increased to 25138 ha or 8.7% of the total area in 2006 and 44418.5 ha or 15.1% in 2021. Urbanization and the need for agricultural land have been identified as driving forces behind these changes and Markov chain analysis reveals future regression of coastal ecosystems such as mangroves, dense forest, swamp, and crop ender palm. These outcomes have far-reaching policy direction of environmental sustainability target in Benin coast.

## 1 Introduction

Land use dynamics are not without implications for the environment and ecosystems, and this is reinforced by the growing population, which is today's main driving force for land-use change (Molla et al., 2015). Major problems such as soil degradation and erosion (Khaledian et al., 2016), desertification (Awotwi et al., 2018), biodiversity loss (Degife et al., 2019), habitat destruction (Rimala et al., 2019; Rodríguez-Martín et al., 2022), and natural processes such as climate change (Froese et al., 2019; Ramadhan et al., 2021; Bekun, 2022) can be linked to land-use change as they alter the ability of natural ecosystem to support and provide the desired services. As a matter of fact, coastal urban areas are the areas of concentration of several economic activities and this favours urban-rural migration (Andrés et al., 2018). Although coastal ecosystems are subject to ongoing destruction due to human activities, studies addressing the issue are sporadic, especially in Africa, South America, and Oceania (Davidson et al., 2014). As land-use change is cited as a major cause of ecosystem loss and landscape fragmentation in coastal areas (Torbick et al., 2006), assessment and monitoring of these changes are needed to measure their impact on ecosystems at spatial and temporal scales. Remote sensing and geospatial techniques now provide tools to assess changes in coastal landscapes (Davidson et al., 2018).

In West Africa and particularly in Benin, studies have been conducted on different themes such as: coastline dynamics (Assogba et al., 2021; Hounguè et al., 2018; Ndour et al., 2018); coastal risk management (Florence de Longueville et al., 2020; Guerrera et al., 2021; Ndour et al., 2018); assessment of vulnerability and perception of risks by coastal populations (Dossou and Gléhouenou-Dossou, 2007); land use change (Padonou et al., 2017). Studies on land use/cover change have been carried out in two coastal cities in Benin (Teka et al., 2012a, Teka et al., 2012b) however, understanding the basis of the patterns and processes driving land-use change throughout all coastal areas in Benin remains to be elucidated. Also the primary processes that induce land transformation cannot be raised by simple monitoring of the evolution in land-use changes (Alo and Pontius, 2008). Indeed, quantitative sufficient and intensive signals from the land-use model are not revealed in conventional methods of matrix change analysis (Huang et al., 2012) while associating patterns of land use change patterns with processes is what is valued (Don De Alban et al., 2019). And this is indeed the originality of this study, which responds to the knowledge gap for the purpose of policy-making. This understanding will enable the development of appropriate strategies for the management of this area by various stakeholders at different levels. Using the intensity analysis method developed by Zakaria Aldwaik and Gilmore Pontius Jr (2012, 2013), the gains and losses of land use categories can be known (Ekumah et al., 2020) as well as the patterns and processes that define these transformations (Bufebo and Elias, 2021). Future modeling is also possible with the Markov chain model to predict land use/cover expansion and change (Padonou et al., 2017).

The objective of this study is to assess land use land cover in the coastal zone of Benin in order to better anticipate the phenomena of loss and fragmentation of ecosystems and to provide guidelines for policy-making.

## 2 Investigation area

**Figure 1:**
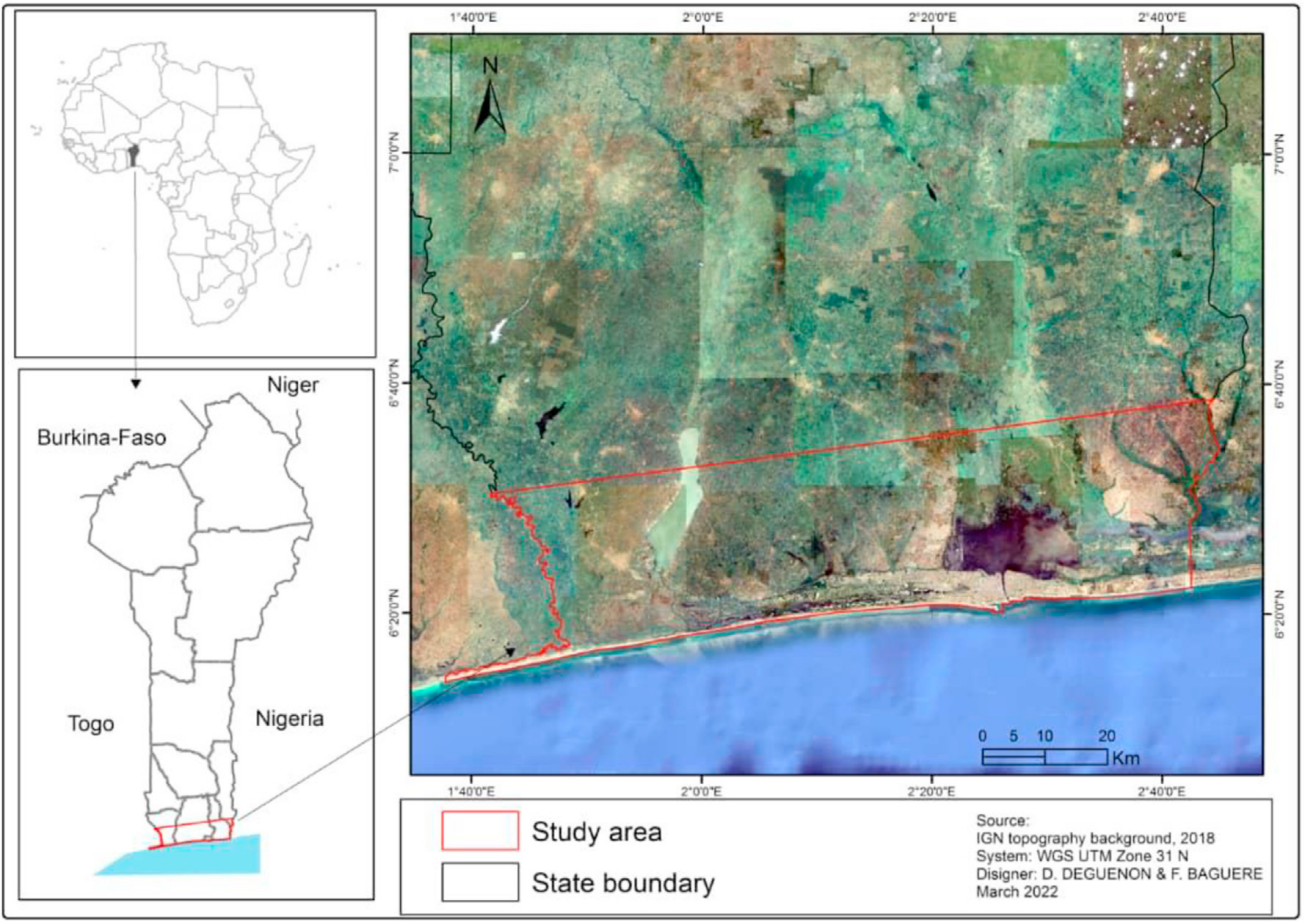
Map showing study area location.

The study area is the Beninese coastline that lies between latitudes 6°10' and 6°40' and longitudes 1°40' and 2°45' (Figure 1). It covers an area of about 12,000 km2, and represents 10.5% of the national area of Benin; it is defined as a lagoon coast because there are ancient and recent lagoons covering an area of more than 30 km. This area abounds in flats of depressions as well as blind drainage areas. Considering the classification of Troll (1965), the Beninese coast is located in the humid tropical summer climate zone (V2). The climate varies from two rainy seasons from April to July and September to November to two dry seasons from August to September and December to March (Adam and Boko, 1993). Basic daily thermal amplitudes of about 33 °C and annual rainfall ranging from 820 mm to 1300 mm characterize this zone (CEDEO, 2013). Several ecosystems such as dry forests, gallery forests, and boundary rainforests are found in the area, marshes, wetlands (listed in Ramsar sites No. 1017 and No. 1018), and the banks of the main rivers (Mono, Ouémé, Couffo), although in a degraded state, are the riches of this zone. Agriculture characterizes the coastal landscape, although the emphasis could also be placed on palm tree vegetation (*Elaeis guineensis*) which dominates the area. In this environment, sandy, ferritic and hydromorphic soils are most present (Adam and Boko, 1993). Thus, the six departments (out of the twelve in Benin) that border the coastline cover a total area of 11,720 km^2^ with a population of 2.7 million inhabitants, representing an average density of 230 inhabitants per km^2^, in contrast to the rest of the country.

More than 2/3 of the population lives in rural areas. The exodus is extremely marked. The urban population is growing at a rate of approximately 7.4% per year, which puts a lot of pressure on the infrastructure and the labour market in the cities. From a socio-economic point of view, the Beninese coastline is the economic lung of Benin, the main cities, port, and airport infrastructures, as well as industrial facilities. Migration to the coastal strip explains the high human density, high growth rates, and demand for land for construction. People are involved in a variety of activities: agriculture, fishing, salt farming, tourism, trade, quarrying, livestock breeding, and processing of agricultural products (Environnement & Climat, 2019).

## 3 Methodological approach

### 3.1 Data collection, processing and classification

The data used in this study are included both primary and secondary data. Primary data included semi structured questionnaire survey, GPS measurements for developing training samples and validation of classification results. Secondary data comprised of Landsat satellite images for the years 1991, and 2021 and Spot satellite image for the year 2006. Also topographic maps, as well Google Earth images were also used. Landsat images were acquired free of charge from the USGS website (https://earthexplorer.usgs.gov/) and Spot from the website (https://regards.cnes.fr/user/swh/modules/60). The spot images were used for the year 2006 for two different reasons. The source of the images used as well as the resolution of the sensor/row path and the dates of acquisition are given in Table 1. Firstly, the Landsat 7 status images have had a sensor failure for some years now. This is the Scan Line Corrector (SLC), an oscillation sensor that creates black bands from the edges of the image to the center (Bernstein, 1976). As a result, linear holes or scratches appear, causing a 22% loss of information of each image received (Scaramuzza et al., 2004) after correction. Secondly, as Spot images have a better resolution than Landsat and are used in the middle of our study period (2006), they make the results more certain on both sides of our study interval.

**Table 1:** Characteristics of images used for land use/land cover change analysis.

Satellite image	Sensor	Path/row	Resolution (m)	Bands used	Acquisition date	Source
Landsat 4	TM	91/55 91/56	30∗30	4,3,2	27/12/1990	USGS 2022
Landsat 5	TM	92/55 92/56	30∗30	4,3,2	10/10/1991	USGS 2022
Landsat 8	OLI_TIRS	91/55 91/56	30∗30	5,4,3	01/07/2021	USGS 2022
Landsat 9	OLI–2_TIRS-2	92/55 92/56	30∗30	5,4,3	23/12/2021	USGS 2022
Spot4	HRVIR2	**-**	20∗20	**-**	12/01/2008	SWH 2022
Spot 5	HRG2-XS	**-**	20∗20	**-**	24/12/2006	SWH 2022

The satellite image bands were stacked and projected in the Universal Transverse Mercator (UTM) projection system (Zone: 30 N, Datum: WGS84) before undergoing supervised classification with the maximum likelihood classifier using ENVI 5.3. Regions of interest (training ROI and control ROI) were selected for each class and year. According to the land use classes defined by (UEMOA, 2010) in the synthesis of the regional diagnosis during the coastline monitoring studies and West African coastal master plan, the study environment was classified into 10 classes: Built-up area, crop under a palm, water, dense forest, mangrove forest, swamp, ocean, a mosaic of crop and out of crop, beach, plantation. QGIS 3.22 and ArcGIS 10.3 software was used for the delineation of the study environment.

The years mentioned above were chosen because they determine specific periods in the history of the coastal zone of Benin. By the 1991s, the notorious consequences of the different phenomena taking place in the coastal zone are increasingly attracting the attention of researchers and decision-makers. The economic pressures were already related to typical coastal activities: fishing, aquaculture, port activities, seaside tourism, increasing urbanization, and developments that often triggered or aggravated erosion phenomena (Pirazzoli, 1993); . The primary sector represents about 43% of Benin's GDP (Prudencio et al., 2002). Among the economic activities that contribute to the degradation of this environment is the cutting of wood for use either as timber or as firewood, which, together with certain cultivation practices, promotes soil erosion. Official statistics indicate that consumption in the coastal zone exceeds 2.2 million tones per year (MEHU, 2019). In addition, a significant part of this wood is taken from the mangrove, occupied by local communities specialized in artisanal salt production. The combination of these different factors has considerably destroyed the mangrove vegetation, even threatening it with extinction (Orekan et al., 2019). After this alarm bell was rung and awareness was increasingly raised, the Beninese government banned the exploitation of marine sand in 2007 and began to plan sea defense in 2008. Unfortunately, the storms of March 2007 subjected the lung of the Beninese economy (Cotonou) to terrible coastal erosion. These consequences are therefore increasingly visible in 2021. The period 1991–2021 covers an interval of 30 years and 2006 represents half of the time.

### 3.2 Data processing

#### 3.2.1 Land use and land cover (LULC) change

Once the image classification was completed, the LULC maps were brought out. The sample drone images, topographic maps, and Google Earth were used to assess the accuracy of the maps. ENVI 5.3 software was used to quantify the changes in the LULC maps.

#### 3.2.2 Intensity analysis

Intensity analysis is a mathematical framework for expressing differences within a set of categories that exist at multiple points in time (Quan et al., 2020). Intensity analysis communicates the changes in terrain in a region during time intervals of various durations. Authors have used Intensity Analysis to analyze land use change where the spatial extent is a single region (Minaei et al., 2018; Yang et al., 2017). Intensity analysis is an accounting framework for structuring interpretation by mathematically and graphically communicating the information contained in transition matrices (Aldwaik and Pontius, 2012, 2013). Many scientists express transitions between categories as Markov matrices (Awotwi et al., 2018; Berlanga-Robles et al., 2011). Markov matrices show ratios that express the same concept as the transition intensities of intensity analysis. However, Markov matrices do not show transition sizes; thus, Markov matrices alone do not explain transitions as intensity analysis does.

The interval level 'Intensity Analysis' compares time intervals in terms of the size and intensity of the gross change in each region (Pontius, 2019).

The Intensity Analysis category level compares the categories in terms of size and intensity of loss and gains in each region during each time interval (Pontius et al., 2013). An annual change in a LULC category is termed 'dormant' when it is less than the uniform intensity of change in a time interval. In contrast, a change is termed active when the annual change in a LULC category is greater than the uniform intensity of change in a time interval.

The transition level 'Intensity Analysis' compares the transitions in terms of size and intensity in each region during each time interval (Pontius et al., 2013). It compares how each category gains other categories during each time interval (Quan et al., 2020). If the observed transition intensity of a particular LULC category is higher than the uniform transition intensity, it means that the gain of that category targets the other category. In contrast, if the observed transition intensity of a particular LULC category is less than the uniform transition intensity, then the gain in that category avoids the other category. The largest gain categories for each time interval were considered for transition analysis in this work.

The Microsoft Excel program freely available on the Intensity Analysis website (https://sites.google.com/site/intensityanalysis/) developed by Safaa Zakaria Aldwaik and Robert Gilmore Pontius Jr. was used. The article by Aldwaik & Pontius (2012) gives further details on the mathematical equations used at each level of the intensity analysis.

#### 3.2.3 Questionnaire survey

To find out what factors influenced the observations made on the satellite images, surveys, interviews with resource persons, and focus group discussions were conducted with stakeholders present in our study area using a structured seed questionnaire incorporated in the KoBoCollect application. Criteria such as age and length of stay in the study area were determined. For this purpose, 55 years of age or older was the required age to answer the questions related to the observed changes and the related forces. The sample size of respondents (n_i_), calculated using the formula from Dagnelie (1998) as 144.06 was rounded to 150.Where U1- α/2 is the value of the normal random variable for a probability of 1-α/2 (for a significance level α = 0.05, U1-α/2 = 1.96); d = 0.08 is the margin of error set taking into account the desired precision and p_i=_ 30/50 = 0.6.

#### 3.2.4 LU/LC future change

These predictions of the changes that will be observed in each land-use class were made using the transition matrices for each year. Considering that the observed changes in each study period remain: 1991–2006, 2006–2021, and 1991–2021, the Markov chain was used for the analysis of future scenarios. This model was validated using a χ2 test (Padonou et al., 2017).

## 4 Results

### 4.1 Classification accuracy assessment

The confusion matrix (Table 2) was used to assess the reliability as well as the precision of the results. Thus, the overall accuracy, producer and user accuracy, and kappa statistics with mathematical precision were determined. From the years 1991, 2006, and 2021, the overall accuracy of the classified images are 73.80; 82.81; 72.06% respectively and the kappa statistic for LU/LC maps is 0.96, 0.94, and 0.92 (Table2). Thus, we infer that classified images and referenced data have a good level of agreement. Table 2 provides details on the ratio of overall accuracy to individual group accuracy of the three classified images.

**Table 2:** Accuracy assessment (in percent) of the 1991, 2006, and 2021 LU/LC maps of Benin Coast.

	1991		2006		2021	
**Land use/land cover**	Producer’s accuracy (%)	User’s accuracy (%)	Producer’s accuracy (%)	User’s accuracy (%)	Producer’s accuracy (%)	User’s accuracy (%)
**Water**	84.38	73.18	84.10	74.88	64.44	74.10
**Ocean**	21.13	23.41	21.03	23.41	73.21	81.51
**Built up area**	80.44	86.36	80.44	83.20	83.20	74.44
**Swamp**	25.25	21.95	25.25	21.95	81.95	85.25
**Beach**	26.19	33.12	22.10	20.00	42.36	58.82
**Plantation**	15.62	33.91	15.62	33.91	73.91	75.62
**Dense forest**	28.33	33.28	28.33	33.28	55.09	59.87
**Mangrove forest**	78.54	76.29	75.40	71.19	81.19	85.40
**Mosaic of crup and out of crup**	82.57	85.62	83.47	85.62	85.62	73.47
**Crup under palm**	86.39	81.18	84.18	82.98	82.98	84.18
**Overall accuracy (%)**	73.80		82.81		72.06	
**Kappa coefficient**	0.96		0.94		0.92	

### 4.2 Land use/land cover change outputs

**Figure 2:**
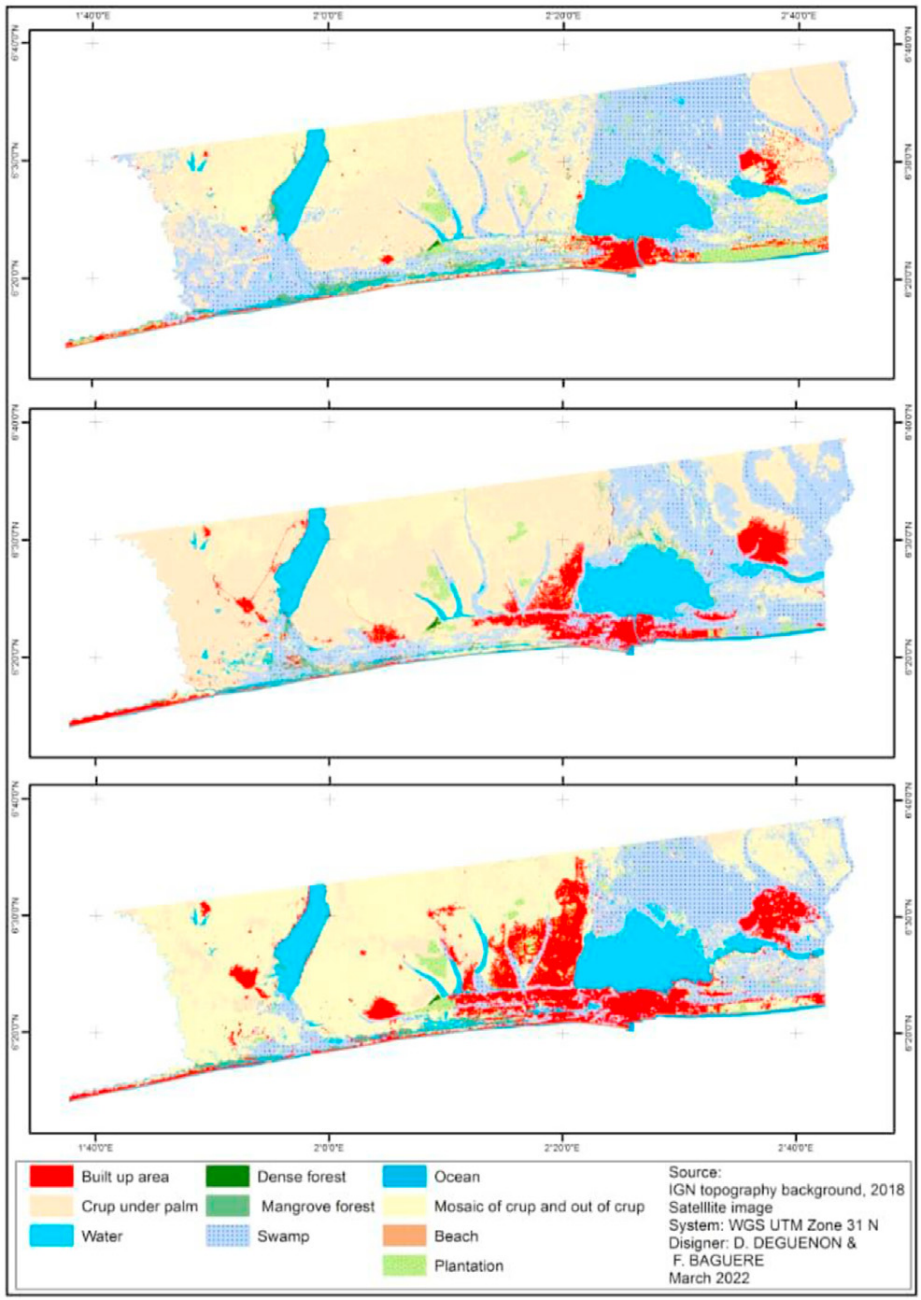
Land use/land cover changes in coastal areas of Benin in the years 1991, 2006 and 2021.

Notable changes in land cover have been observed in our study area over the past three decades. Figure 2 below show the land use maps in 1991 2006 and 2021 and the changes that occurred during these periods. From 1991 to 2021 through 2006, there has been a conversion of agricultural land (mosaic of crop and out of crop, crop ender palm) into a built-up area. Also, the loss of forest cover is part of the visible evidence of change observed. The statistics on observed changes are further detailed in Table 3.

**Table 3:** Transition matrix (ha) for coastal zone of Benin for 1991–2006, 2006–2021 and 1991–2021.

1991												
	CLASS_NAME	Built up area	Crup under palm	Water	Dense forest	Mangrove	Swamp	Ocean	Mosaic of crup and out of crup	Beach	Plantation	Total
2006	Built up area	8722.2	1768.4	508.2	2.6	495.7	2647.8	0.2	8803.4	85.0	2104.7	25138.2
	Crup under palm	468.0	53119.5	1014.6	93.0	2535.0	28508.9	0	39947.5	3.5	2470.0	128159.9
	Water	87.3	670.8	27926.2	2.7	122.0	1205.2	5.4	220.4	36.8	82.1	30358.8
	Dense forest	0	17.6	0	101.3	0	0	0	4.0	0	44.7	167.6
	Mangrove	114.0	302.3	420.8	0.3	773.4	2839.8	0.0	485.9	2.0	429.5	5368.1
	Swamp	952.9	10170.8	1023.1	20.4	1751.0	26757.3	0.5	4299.6	39.4	3600.8	48615.8
	Ocean	86.2	0.0	21.1	0.0	1.7	0.0	1407.6	2.5	787.4	243.8	2550.1
	Mosaic of crup and out of crup	767.7	18271.6	1035.8	130.4	921.3	9100.3	0	17077.6	8.1	1398.4	48711.1
	Beach	315.6	1.6	28.3	0	0.7	1.3	135.1	19.0	852.7	118.7	1473.0
	Plantation	29.1	463.8	0	13.1	24.8	132.5	0.0	270.4	0.9	2011.4	2945.8
	Total	11543.0	84786.4	31978.1	363.6	6625.6	71193.1	1548.7	71130.3	1815.7	12504.0	293488.5
	CLASS_NAME	Built up area	Crup under palm	Water	Dense forest	Mangrove	Swamp	Ocean	Mosaic of crup and out of crup	Beach	Plantation	Total
2021	Built up area	19198.6	13504.4	1232.4	1.7	650.7	3849.6	15.9	5322.9	372.8	269.5	44418.5
	Crup under palm	40.1	17219.6	21.6	3.6	189.2	3601.5	0	6177.7	0.2	152.9	27406.5
	Water	730.7	1989.2	28015.6	0	663.2	2221.9	10.1	1730.1	9.8	0	35370.5
	Dense forest	0	8.0	0.1	103.2	0	4.1	0	0.8	0	0	116.2
	Mangrove	148.2	1116.6	219.5	0	497.9	348.7	0.4	197.8	4.1	0	2533.4
	Swamp	254.8	15098.5	583.9	0	2058.9	27517.5	4.0	5981.0	22.3	60.2	51581.1
	Ocean	46.7	2.8	42.6	0	1.2	0.4	2304.3	2.9	157.9	0.6	2559.3
	Mosaic of crup and out of crup	963.4	78255.1	793.4	47.0	107.0	10741.5	0.2	28275.9	4.4	732.8	119920.5
	Beach	194.4	14.9	25.1	0	10.5	33.2	263.7	23.4	927.5	18.7	1511.4
	Plantation	196.1	3420.9	30.8	15.3	295.4	1278.6	1.9	1059.3	2.7	1769.9	8071.1
	Total	21772.9	130629.8	30965.0	170.9	4473.9	49597.2	2600.6	48771.7	1501.8	3004.7	293488.5
1991												
	CLASS_NAME	Built up area	Crup under palm	Water	Dense forest	Mangrove	Swamp	Ocean	Mosaic of crup and out of crup	Beach	Plantation	Total
2021	Built up area	9082.0	9089.1	1371.1	49.0	1298.3	5139.9	2.0	15506.4	290.5	2827.0	44655.2
	Crup under palm	90.8	11773.4	44.3	23.2	302.1	5810.7	0	8595.9	0	573.8	27214.2
	Water	115.4	946.1	28558.9	0.5	697.0	3390.8	4.8	577.4	12.0	378.7	34681.6
	Dense forest	0	16.5	0	101.8	0	0	0	0.4	0	10.1	128.7
	Mangrove	76.2	35.1	484.3	0.2	1080.9	725.5	0	200.1	2.8	272.3	2877.3
	Swamp	913.1	7993.2	959.9	89.6	1577.6	34266.0	0.0	3979.2	34.8	2514.0	52327.4
	Ocean	107.3	0	28.9	0	3.2	0	1344.9	4.4	763.9	257.1	2509.7
	Mosaic of crup and out of crup	748.3	53322.9	466.3	80.0	1196.3	19763.9	0	41090.1	0.8	3018.3	119686.8
	Beach	241.5	4.4	44.1	0	16.0	0	197.0	10.7	710.2	258.6	1482.5
	Plantation	36.1	1620.5	41.4	19.5	448.5	2121.2	0	1238.9	0.8	2398.2	7925.1
	Total	11410.7	84801.2	31999.1	363.9	6619.8	71217.9	1548.7	71203.4	1815.7	12508.0	293488.5

### 4.3 Transition matrices between 1991, 2006, and 2021 in Benin coast

Table 3 represents the transition matrix between different land-use patterns in the coastal zone of Benin. The numbers on the diagonal (in bold) indicate stability.

The changes observed during our study period do not follow any chronology. They vary from one period to another and from one class to another. Nevertheless, the classes listed have remained over time although there are large oscillations within them. During the period 1991–2006, fields under palm trees were the largest land use with 28.9% of the total area. This occupation will decrease over time to 22.7% in 2006 and 7.2% of the total area in 2021. Classes such as the mosaic of crop and out of crop and swamp, dense forest have undergone the same trend while the opposite trend is observed in built-up areas. From 3.9% of the total area in 1991, this class has increased to 8.7% of the total area in 2006 and 14.8% in 2021.

### 4.4 Intensity analysis

#### 4.4.1 Interval level

**Figure 3:**
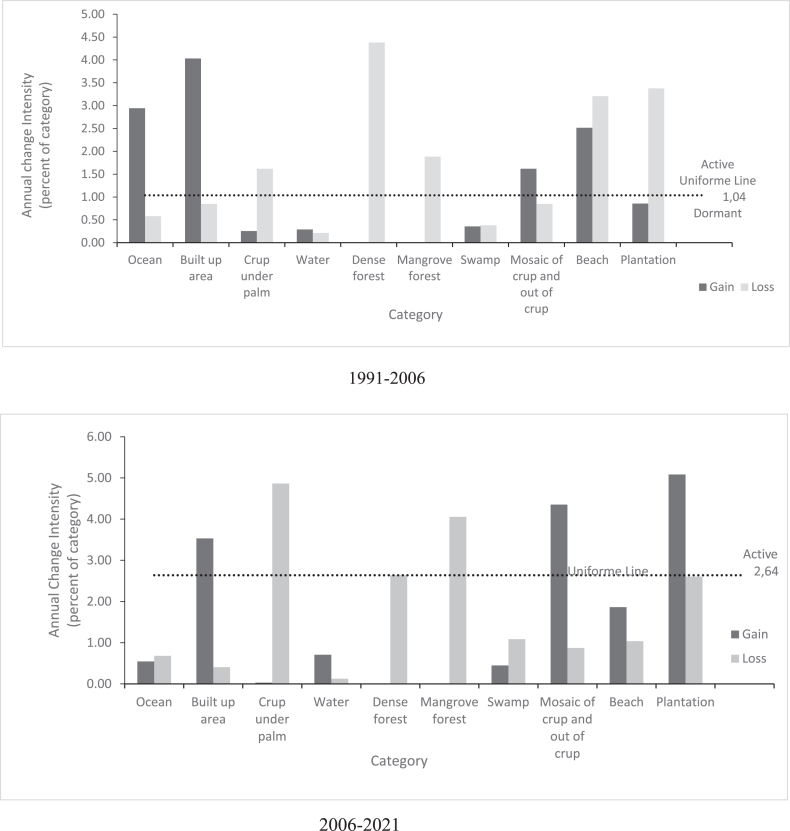
Interval level Intensity Analysis results for 1991–2006 and 2006–2021.

The analysis of interval intensity in our study area (Figure 3) reveals that annual changes in the second time interval (2006–2021) were faster (2.64%) compared to the other two intervals. 1991–2006 was the time interval with the lowest annual change (1.04%).

#### 4.4.2 Category level

**Figure 4:**
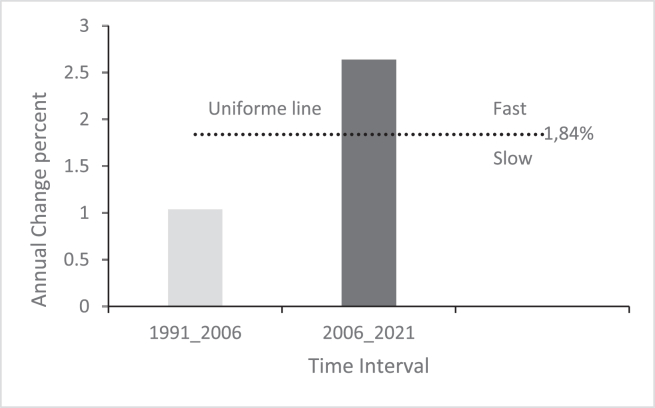
Category level Intensity Analysis showing active losing and gaining categories in Benin coast for 1991–2006 and 2006-2021time intervals.

The results of the intensity analysis at the category level in the three-time intervals of our study area are shown in Figure 4. During the first-time interval (1991–2006), dense forest and mangrove forest experienced only active loss while built-up areas and the ocean were the classes that experienced the most active gains. There was not much difference in the inland water class as the losses and gains in this class remained dormant.

In the second time interval 2006–2021, the situation of dense forests and mangrove forests remained relatively unchanged while plantations had active gains. Built-up areas always had active gains and dormant losses. Mosaic crop and out of crop had active gains while crop under palm had active losses.

#### 4.4.3 Transition level

**Figure 5:**
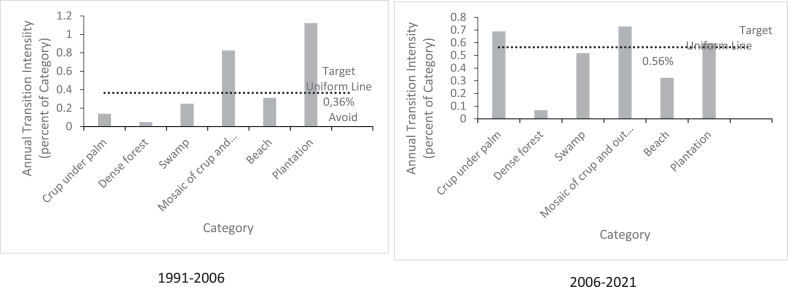
Transition to built-up area on Benin coastal zone for 1991–2006 and 2006–2021 time intervals.

For the results of the third part of the intensity analysis (transition level), the classes with the largest gains were taken into account; these are built-up areas, mosaic crop and out of crop, ocean and plantations. Figure 5 shows that the increase in built-up areas during the first time interval is due to the destruction of the mosaic crop and out of crop and plantations, while the destruction of the crop under the palms is added during the second time interval.

**Figure 6:**
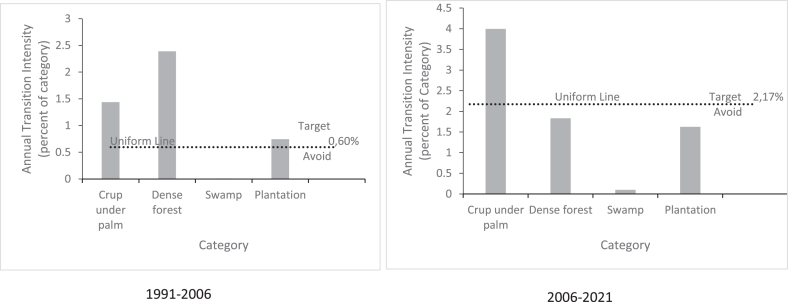
Transition to mosaic of crup and out of crup (cultivated area) on Benin coastal zone for 1991–2006 and 2006–2021 time inetervals

The increase in cultivated area is due to the destruction of forest, crop under a palm, and part of the plantations between 1991-2006, but between 2006 and 2021 this increase is mainly due to the regression of crop under palm (Figure 6).

**Figure 7:**
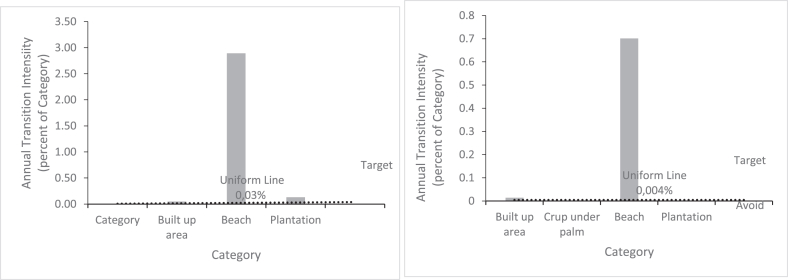
Transition to ocean on Benin coastal zone for 1991–2006 and 2006–2021 time intervals.

**Figure 8:**
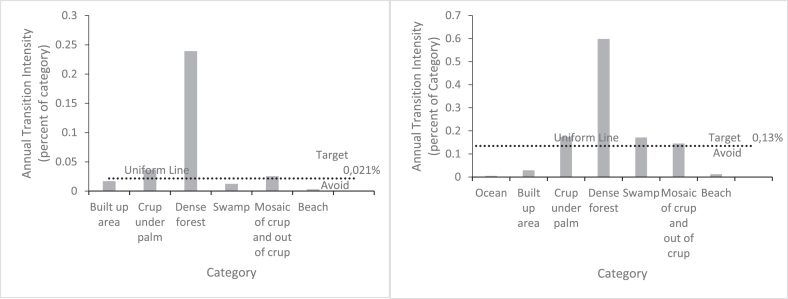
Transition to plantation on Benin coastal zone for 1991–2006 and 2006–2021 time intervals.

As for the increase in ocean area, Figure 7 shows that in both time intervals it is due to the loss of shoreline land. Figure 8 explains the gain in plantations. The increase in plantation area is due to the loss of dense forest.

### 4.5 Driving forces of LU/LC change in Benin coastal area

**Figure 9:**
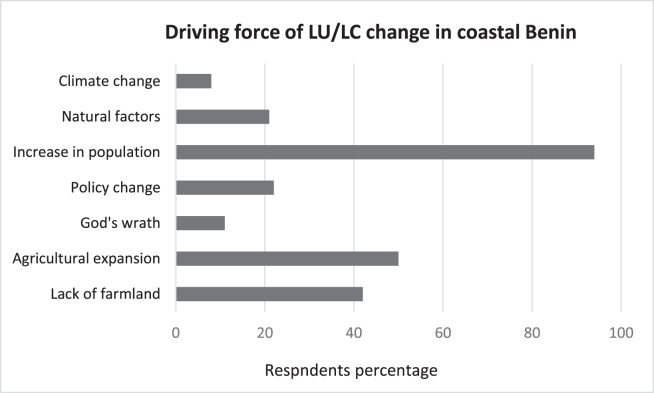
Driving forces of land-use change based on the population understanding.

According to the interviewees, population increase is the main cause of the decline in forest cover and different coastal ecosystems over the years. Factors such as lack of farmland, agricultural expansion, and policy changes are part of the driving forces leading to this situation (Figure 9).

### 4.6 Future scenarios for LU/LC using Markov chain analysis

- The map area of the year 2006 and that expected from the modeling of the period 1991–2021: X-squared = 0.37376, df = 9, p-value = 1 > 0.05;
- The map area of the year 2021 and that expected from the modeling of the period 1991–2006: X-squared = 0.15897, df = 9, p-value = 1

The model can therefore simulate the evolution of LULC.

**Figure 10:**
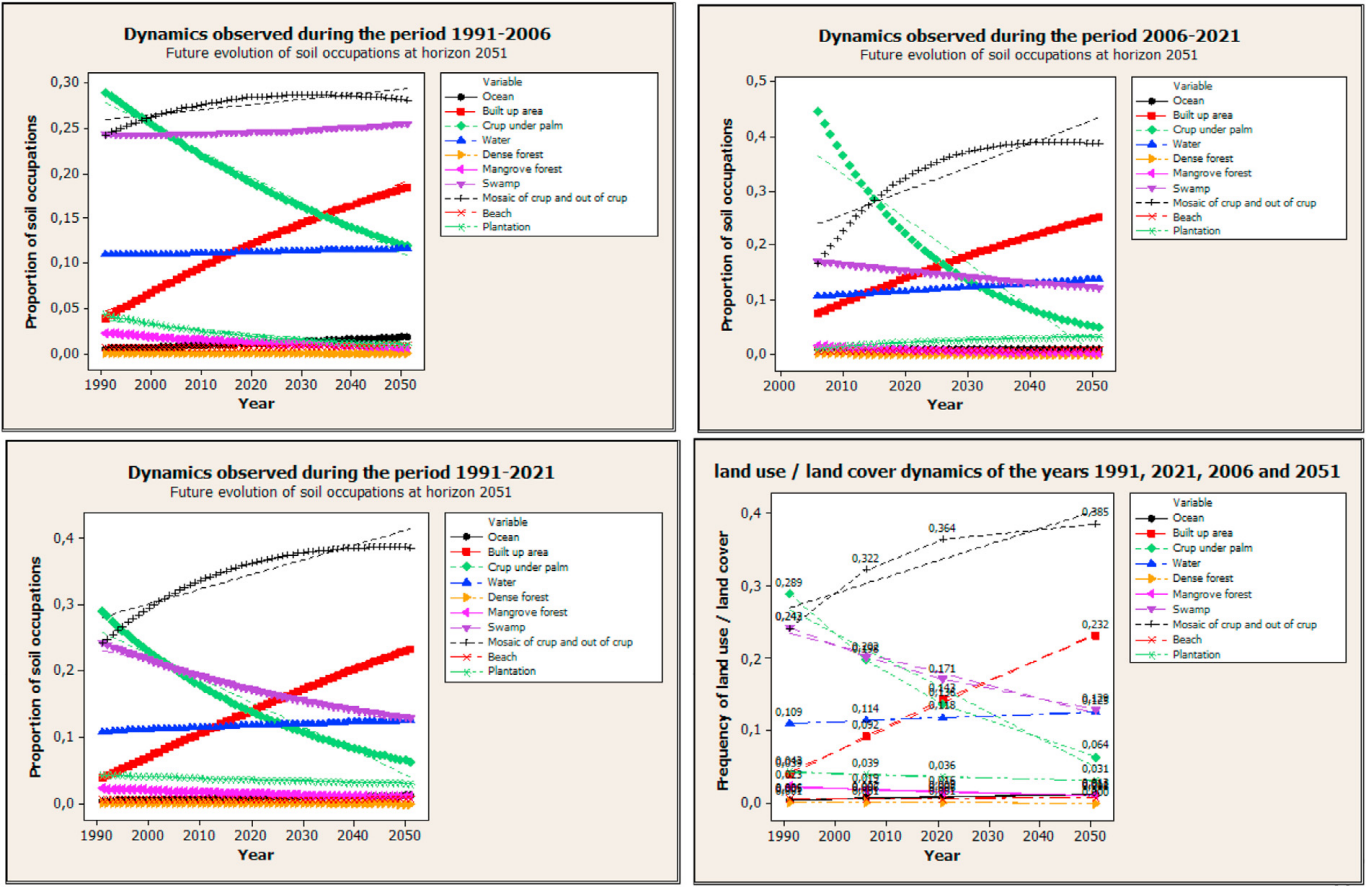
Future evolution of soil occupation at horizon 2051 using three future scenarios.

**Figure 11:**
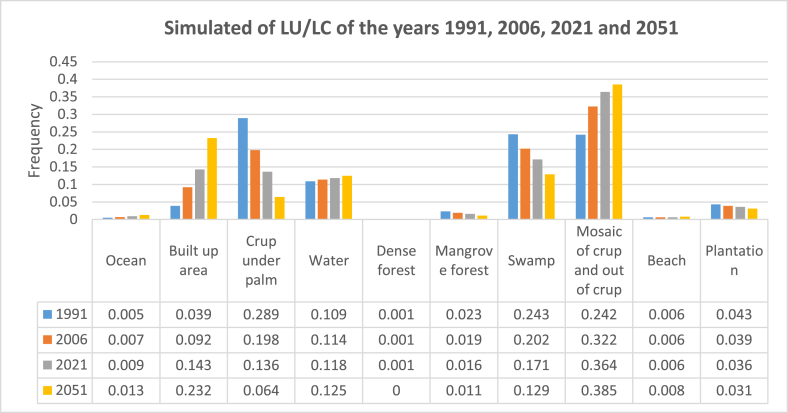
Future evolution of soil occupation at horizon 2051 using three future scenarios.

Figures 10 and 11 show the future predictions for 2051 taking into account the dynamics observed during the periods 1991–2006, 2006–2021, and 1991–2021.

Taking into account the dynamics observed during the first period (1991–2006) to model the horizon 2051, the area of crop under a palm, dense forest, mangrove forest, plantation, and the swamp will decrease respectively by 28.9%, 0.01%, 2.3%, 4.3%, and 24.3% while built-up area, a mosaic of crop and out of crop, beach, water, and ocean will gain land respectively by 3.9%, 24.2%, 0.6%, 10.9%, and 0.5%.

The dynamics observed during the second (2006–2021) and third (1991–2021) time intervals predict that in 2051 the same trend of decrease or increase in the different classes will be observed but with different percentages.

## 5 Discussion

Intensity analysis and the Markovian chain model were used in this work to map the Spatio-temporal dynamics of land use patterns over 30 years and predict future transformations for each land-use class. This work was carried out in coastal zone, which contains several ecosystems such as dry forests, gallery forests and boundary wet forests, marshes, wetlands (listed in Ramsar sites no. 1017 and no. 1018), and the banks of the main rivers (Mono, Ouémé, Couffo). Carried out over two-time intervals: 1991–2006 and 2006–2021, the results reveal that the land use classes have remained the same although there have been changes within these classes over time. It could be said that the driving forces responsible for these changes have increased over time, as more pronounced changes can be observed in the second time interval. These changes could be related to anthropogenic activities, as the interpretation of the land use maps shows a clear increase in the classes related to anthropogenic activities. This is confirmed by the fact that, according to 98% of the respondents, rapid urbanization in the study area is one of the driving forces behind the changes observed over time. This confirms the results of Teka et al., 2012a, Teka et al., 2012b who pointed out that the coastal zone of Benin is shelter to about 60% of Benin's population on 8% of the country's total area.

The demographic and urban dynamics of the coastal zone are worrying, particularly in the five Communes (Sèmè-Podji, Cotonou, Abomey-Calavi, Ouidah, and Grand-Popo) that open onto the sea, where the viable space for urbanization is predominantly made up of low-lying coastal strips threatened by risks of coastal erosion and flooding. According to Faustine et al. (2020) the direct consequence is overexploitation of natural resources followed by various forms of environmental pollution.

The intensity analysis using the transition matrix indicates the movement of cover classes in terms of gain, loss, and major transitions of different LULC categories. Built-up areas, mosaic of crop and out of crop, the ocean had gains in both time intervals while classes such as dense forest, mangroves, and plantations regressed. The extension of built-up areas comes mainly from crop under the palm and somewhat from swampy areas. This suggests that poor farming techniques have led to the saturation of the land under the palm groves, which has given way to construction. In the second period, the loss of certain natural formations such as mangroves and dense forests can be observed.

According to DGEC (2020) national report indicated that migration to the coastal strip explains the high human density, high growth rates, and demand for agriculture land and for construction land. From 2,164,184 inhabitants in 1989, it increased at a rate of 0.22 in 1999 to a rate of 0.66 in 2012. As a result, cities are expanding towards the peripheries with informal settlements, large building construction, and the taking over of uninhabited areas and the coastal strip.

Coastal erosion is noticeable in different parts of the coastal zone. At the border with Togo (from Hilla-Condji to Agoué), the coast showed a tendency to sedimentation until 1985. However, from that time until 1991, erosion began. This is certainly due to the decrease in sedimentary input from the Togolese coastline as a result of protection. In the Grand-Popo area, the city appears to be a sensitive point with periodic fluctuations. But the real identification of the causes remains to be done. Since the great upheavals studied by Henri et al. (2002) and the successive episodes that followed leading to the first destruction of the city, the sea has advanced continuously until 1982 and continues to this day. As for the mouth of the Mono river, the situation has changed considerably since the commissioning of Nangbeto in 1988. Indeed, the Nangbéto dam releases generate strong flushing currents responsible for the spectacular erosion observed at the mouth since 1990, resulting in the swallowing of more than 50% of the riparian villages and the narrowing of the fishing route at this level by a thin strip of land (SDAL, 2020). Faced with such a situation, so far, the measures implemented such as breakwaters, sea-front walls, protection of the upper beach in rock or gravel, the groins perpendicular to the coast, either in rock, in gabions, or metal sheet piles, or in wood, concrete for the protection of our coasts have been relatively effective. Several projects of the coastal zone management dealing with firewood, reforestation, the Cotonou Autonomous Port project, and the PGRN, have had positive impacts. This can be seen in the fertilization at certain levels of the coast, the establishment of plantations on the periphery of the coast, and also in the various communes of the coastal zone to deal with the problem of uncontrolled deforestation.

Despite the efforts observed at the level of the various actors in charge of the management of the coastal zone, the modeling of the dynamics of land use by 2051 taking into account the past scenarios do not yet show a great restoration. The predictions show that by 2051 there will be practically no more dense forest in the coastal zone of Benin, but this can still be discussed depending of actions which can be put in place.

## 6 Conclusion and policy recommendation

Using the intensity analysis method and the Markov chain model, this study used remote sensing and field survey data to map the Spatio-temporal dynamics of land use patterns over 30 years, the underlying driving forces, and future predictions of these current occupations.

From 1991 to 2021, through 2006, there has been a conversion of agricultural land (mosaic of crop and non-crop, crop ender palm) and forest into built-up areas. Urbanization is one of the driving forces responsible for the different transformations observed in our study. Other phenomena such as coastal erosion are part of the imminent problems facing the coastal zone of Benin although actions such as sea defense, and coastal zone management projects continue to be implemented by the authorities at various levels. As a perspective, these results are important for assessing the impact of sea-level rise on different coastal ecosystems to contribute to Integrated Coastal Zone Management in Benin.

The originality of this study is that it uses a mathematical framework to communicate changes in terrain in a region over time intervals. This study provided relevant results on the dynamics of land use land cover over the last 30 years and future scenarios on Benin coast. However, is still to be done. Limitation of the work is that the study did not take into account the complete delineation of the coastal zone as defined in the country's texts. This is due to the fact that apart from the satellite images used, field surveys were carried out and due to limited resources, the study covered 30 km from the coast to the mainland.

Based on the above outcome, a number of policy recommendation are summarized:

1 Establish a system and tools to monitor internal and external migration in the coastal zone
2 Implement the text on the attributes assigned to each soil type in the coastal zone of Benin
3 Organize bi-annual exchange workshop on challenges and achievements between all stakeholders in the area
4 Fund research for continuous evaluation of the impact of measures taken in order to adjust them.

## Declarations

### Author contribution statement

Sèna Donalde Dolorès Marguerite DEGUENON, PhD: Conceived and designed the experiments; Performed the experiments; Analyzed and interpreted the data; Contributed reagents, materials, analysis tools or data; Wrote the paper.

O. N. Fabrice BAGUERE: Analyzed and interpreted the data; Contributed reagents, materials, analysis tools or data.

Oscar TEKA; Denis Worlanyo Aheto; Brice A. SINSIN: Conceived and designed the experiments; Contributed reagents, materials, analysis tools or data.

### Funding statement

Ms. Sèna Donalde Dolorès Marguerite DEGUENON was supported by 10.13039/501100005920Organization for Women in Science for the Developing World (OWSD_SIDA) [3240314466].

### Data availability statement

Data included in article/supp. material/referenced in article.

### Declaration of interest’s statement

The authors declare no competing interests.

### Additional information

No additional information is available for this paper.
